# Retinal nerve fiber layer thickness analysis in suspected malingerers with optic disc temporal pallor

**DOI:** 10.4103/0301-4738.55077

**Published:** 2009

**Authors:** Mustafa Civelekler, Ismail Halili, Faith C Gundogan, Gungor Sobaci

**Affiliations:** Department of Ophthalmology, Gulhane Military Medical Academy, Ankara, Turkey

**Keywords:** Malingering, nerve fiber layer, optic disc temporal pallor, visual acuity

## Abstract

**Purpose:**

To investigate the value of temporal retinal nerve fiber layer (RNFL_temporal_) thickness in the prediction of malingering.

**Materials and Methods:**

This prospective, cross-sectional study was conducted on 33 military conscripts with optic disc temporal pallor (ODTP) and 33 age-and sex-matched healthy controls. Initial visual acuity (VA_i_) and visual acuity after simulation examination techniques (VA_aset_) were assessed. The subjects whose VA_aset_ were two or more lines higher than VA_i_ were determined as malingerers. Thickness of the peripapillary RNFL was determined with OCT (Stratus OCT™, Carl Zeiss Meditec, Inc.). RNFL_temporal_ thickness of the subjects were categorized into one of the 1+ to 4+ groups according to 50% confidence interval (CI), 25% CI and 5% CI values which were assessed in the control group. The VAs were converted to LogMAR-VAs for statistical comparisons.

**Results:**

A significant difference was found only in the temporal quadrant of RNFL thickness in subjects with ODTP (*P*=0.002). Mean LogMAR-VA increased significantly after SETs (*P*<0.001). Sensitivity, specificity, positive and negative predictive values of categorized RNFL_temporal_ thickness in diagnosing malingering were 84.6%, 75.0%, 68.8%, 88.2%, respectively. ROC curve showed that RNFL_temporal_ thickness of 67.5 μm is a significant cut-off point in determining malingering (*P*=0.001, area under the curve:0.862). The correlations between LogMAR-VAs and RNFL_temporal_ thicknesses were significant; the correlation coefficient for LogMAR-VA_i_ was lower than the correlation for LogMAR-VA_aset_ (r=−0.447, *P*=0.009 for LogMAR-VA_i_; r=−0.676, *P*<0.001 for LogMAR-VA_aset_).

**Conclusions:**

RNFL_temporal_ thickness assessment may be a valuable tool in determining malingering in subjects with ODTP objectively.

Functional visual loss (FVL) manifested as visual acuity (VA) loss is one of the most common complaints encountered in ophthalmic practice.[1–6] Many clinical simulation examination tests (SETs) have been described;[7–13] however, it still needs objective criterion.

Optic disc temporal pallor (ODTP) is a fundoscopic appearance characterized by loss of its pink color. Causes may vary (multiple sclerosis, trauma, autosomal dominant optic atrophy, ischemic optic neuropathy, and retinitis pigmentosa), and diagnosing malingering is more troublesome for these patients.

Optical coherence tomography (OCT) is a reproducible and reliable method for assessment of the retinal nerve fiber layer (RNFL).[14,15] Some studies showed relationship between temporal RNFL (RNFL_temporal_) thickness and VA levels.[16–19]

The aim of the study was to determine the value of OCT-assessed RNFL_temporal_ thickness in prediction of malingering.

## Materials and Methods

This study was performed at the tertiary-referral hospital between May 2007 and February 2008. Due to nature of the patient population, malingering is always considered among the differential diagnosis of unexplained visual loss at this hospital.

The research followed the tenets of Declaration of Helsinki; IRB approval and informed consents were obtained after explanation of the study. In this study, 33 military conscripts (33 eyes) with ODTP were selected from 199 subjects suspected of malingering or exaggerating visual loss at presentation to the hospital. At presentation, these 33 subjects had fundoscopic appearance of ODTP in one or both eyes, and a complaint of unilateral VA loss which could not be explained on the basis of thorough clinical examination(s) including color vision and visual field tests. Those eyes having ODTP with full vision were not included in this study group. In order to exclude organic causes of the optic disc, fundus fluorescein angiography (FFA), ultrasonography (US), pattern visually evoked potentials (PVEP) testing and color doppler imaging (CDI) were performed when needed; subjects were referred to a neurologist or psychiatrist, and advanced laboratory techniques including MRI were performed when indicated. Diagnosis of ODTP was made if the two authors’ (CM, GF) common decision was confirmed by the experienced retinal specialist (SG) who was masked to the clinical findings of the case with suspected malingering. Age- and sex-matched 33 healthy military conscripts who had no ophthalmic pathology comprised the control group.

All the subjects were male enlisted soldiers between 19 and 27 years old. Mean age was 23.9±3.9 and 24.0±4.6 years in subjects with ODTP and control groups, respectively (*P*=0.927, not shown in the Table). Spherical equivalent refractive errors varied between -3.25 to +3.50 diopters (D) (not shown in the Table). Individual data of the subjects with ODTP are shown in Table 1.

**Table:** Individual date of the subjects with optic disc temporal pallor

Patient	Visual acuity		Clinical	RNFL_temporal_		OCT diagnosis	Evaluation
				
No.	Initial	After SETS	diagnosis	Thickness	Group		
1	0.1	0.1	Nonmalingering	50	4+	Nonmalingering	True negative
2	0.05	0.1	Nonmalingering	65	3+	Nonmalingering	True negative
3	0.4	0.8	Malingering	77	1 +	Malingering	True positive
4	0.3	0.6	Malingering	74	2+	Malingering	True positive
5	0.2	0.6	Malingering	90	1 +	Malingering	True positive
6	0.2	0.2	Nonmalingering	80	1 +	Malingering	False positive
7	0.2	0.8	Malingering	79	1 +	Malingering	True positive
8	0.2	0.3	Nonmalingering	71	2+	Malingering	False positive
9	0.1	0.7	Malingering	79	1 +	Malingering	True positive
10	0.01	0.6	Malingering	85	1 +	Malingering	True positive
11	0.05	0.05	Nonmalingering	63	3+	Nonmalingering	True negative
12	0.2	0.3	Nonmalingering	74	2+	Malingering	False positive
13	0.01	0.01	Nonmalingering	67	3+	Nonmalingering	True negative
14	0.01	0.01	Nonmalingering	47	4+	Nonmalingering	True negative
15	0.1	0.5	Malingering	60	3+	Nonmalingering	False negative
16	0.1	0.2	Nonmalingering	67	3+	Nonmalingering	True negative
17	0.02	0.02	Nonmalingering	47	4+	Nonmalingering	True negative
18	0.3	0.4	Nonmalingering	59	3+	Nonmalingering	True negative
19	0.4	0.9	Malingering	88	1 +	Malingering	True positive
20	0.2	0.8	Malingering	76	2+	Malingering	True positive
21	0.1	0.2	Nonmalingering	47	4+	Nonmalingering	True negative
22	0.3	0.7	Malingering	77	1 +	Malingering	True positive
23	0.1	0.1	Nonmalingering	74	2+	Malingering	False positive
24	0.05	0.05	Nonmalingering	62	3+	Nonmalingering	True negative
25	0.1	0.5	Malingering	83	1 +	Malingering	True positive
26	0.4	0.8	Malingering	68	2+	Malingering	True positive
27	0.1	0.1	Nonmalingering	61	3+	Nonmalingering	True negative
28	0.01	0.01	Nonmalingering	52	4+	Nonmalingering	True negative
29	0.01	0.01	Nonmalingering	57	4+	Nonmalingering	True negative
30	0.1	0.2	Nonmalingering	59	3+	Nonmalingering	True negative
31	0.05	0.1	Nonmalingering	63	3+	Nonmalingering	True negative
32	0.4	0.5	Nonmalingering	77	1 +	Malingering	False positive
33	0.1	0.4	Malingering	63	3+	Nonmalingering	False negative

SETs: Simulation examination techniques; OCT: Optical coherence tomography; RNFL_temporal_Temporal retinal nerve fiber layer thickness

Routine ophthalmic examinations, including initial best-corrected Snellen acuity (VA_i_), pupillary reactions, biomicroscopy, indirect ophthalmoscopy, and eye movements were performed. The suspicion of malingering was based on the discrepancies between VA_i_ and clinical findings. After VA_i_, best-performed VA or VA_aset_ (visual acuity after simulation examination techniques: SETs) was assessed after certain technical procedures and behavioral observations, which use fogging, dissociation, and fixation techniques employed for detection of malingering included pupillary light reflexes, grimacing in front of the subject,[13] prism dissociation test,[7,8] polarizing lenses,[9,10] distance test,[6,9] bar-reading test (Javal-Cuignet),[6,9] two-perpendicular cylinder test,[6] Harlan test,[6,9] VA repetition test,[6,16,11] hand shaking test,[6] and a new optotype chart introduced by Mojon *et al*.[12] VA_i_ and the VA_aset_ were checked by the experienced clinician (SG) unaware of the VA_i._, and included in the study if the same results were obtained. Any positive result of SETs was accepted enough to reveal malingering. Some of the SETs test only the presence or absence of malingering, but have nothing to do with VA, while others test VAs.

After dilatation with 1% tropicamide (Tropamid^®^, Bilim Co., Turkey), the OCT device (OCT Stratus™, Carl Zeiss Meditec) with RNFL thickness software (version 4.0) were used to acquire three successive circular 3.4 mm diameter scans centered on the optic disc for each eye's RNFL measurement (fast RNFL thickness protocol) during the same hours of the day. The RNFL thickness was automatically assessed by the computer using the algorithm to identify the anterior and the posterior margins of the band of reflectance representing the RNFL, marking the margins with 2 white lines in the visual pathway. Throughout the scanning, the subject kept the eyes constantly fixed on an internal target provided by the equipment. Scans were performed by the same experienced OCT technician who was masked to the clinical status of the subject.

The mean of the data was used to express RNFL thickness as a single average value for the whole 360-degree scan (RNFL_average_) and also as RNFL quadrants (superior, nasal, inferior, temporal). The data obtained in the temporal quadrant (316°-45°) was identified as RNFL_temporal_. It was taken to evaluate the temporal fiber, in which the papillomacular bundle fibers are included.

**Figure 1:**
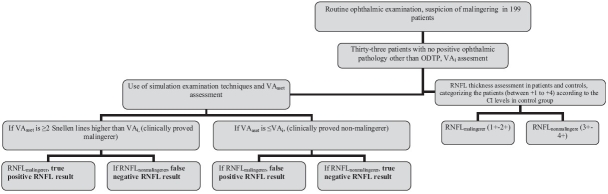
The flowchart representing the study design

In comparing VA_i_ and VA_aset_, each subject was classified as a malingerer or nonmalingerer. If the difference between VA_i_ and VA_aset_ was ≥ 2 Snellen lines, that subject was classified as malingerer. In this study, we used the SETs as the gold standard to prove malingering. From the statistical point of view, an abnormal test indicates a positive result. However, in this study, an abnormal OCT (or a thin RNFL_temporal_) result indicates a negative result indicating the absence of malingering. OCT's sensitivity and specificity to diagnose malingering, and predictive values of positive and negative results were calculated. The study design is shown in Fig. 1.

Snellen acuities (SA) were converted to the logarithm of the minimum resolution of angle acuity (LogMAR VA) for statistical analysis (LogMAR VA=-log_10_ SA). The data were calculated as mean values ±1 SD. The differences between control and malingerers were statistically evaluated with independent samples t test or chi-squared test. In the statistical analysis, *P*<0.05 was considered significant. The statistics were analyzed using SPSS 10.0 (Statistical Package Program for Windows, SPSS Inc., Chicago, Illinois, USA).

## Results

At presentation, all had unilateral VA loss. No organic cause, neither ocular (including anisometropic ambliopia) or systemic origin could be defined in subjects with ODTP in this study group. Similar distributions in refractive errors were observed in the groups (not shown in the Table). Ischiara test revealed 2 of 33 (6%) cases with red-green abnormality. Visual field test (Humphrey^®^ Field Analyzer, Carl Zeiss Meditec) showed constriction of the visual field in the involved eyes of 3 of 33 (9%) cases. None of the subjects in the study group had relative afferent pupillary defect (RAPD). There were 8 of 33 (24%) subjects with bilateral involvement. Among them, abnormalities in latency and amplitude of P100 wave were noted in both eyes of 5 of 33 (15%) subjects. There was no abnormal PVEP value for the rest of the group including 3 of 33 (9%) subjects with bilateral involvement. In medical history, a common complaint of blunt ocular/orbital trauma was present in 13 of 33 (39%) subjects and childhood meningitis in 5 of 33 (15%). Except 3 of 33 (9%) subjects who had SAs of 0.4, 0.6, and 0.7 in the uninvolved eyes which had corneal opacities, 30 of 33 (91%) subjects had SA of 1.0 in the uninvolved eyes. Most (27 of 33 subjects; 81%) subject with bilateral ODTP also had a complaint of visual loss in the right eye. No abnormality was detected in subjects undergone US or FA testing. After SETs, 5 of 33 (15%) cases confessed that they were conscious of ODTP before presentation to our hospital.

When compared to the RNFL thickness values in the control group, there was a significant reduction only in the RNFL_temporal_ thickness in subjects with ODTP [Table 2].

**Table 2:** Retinal nerve fiber layer thickness in subjects with optic disc temporal pallor and control subjects

	RNFL_superior_	RNFL_nasal_	RNFL_inferior_	RNFL_temporal_	RNFL_average_
ODTP	131.5±13.8	92.8±16.1	129.2±22.4	67.9±12.0	105.4±13.0
Control	137.4±16.0	90.9±19.3	134.3±18.6	78.8±14.6	110.3±9.6
p	0.117	0.669	0.316	0.002	0.081

ODTP: optic disc temporal pallor, RNFL: retinal nerve fiber layer, p: independent samples t test

Fifty percent confidence interval (50% CI), 25%CI, and 5%CI values of RNFL_temporal_ in the control group were 77.0 μm, 68.0 μm, and 58.0 μm, respectively. The subjects with ODTP were distributed into one of four groups according to their RNFL_temporal_ values. If RNFL_temporal_ was thicker than 50%CI value that was assessed in the control group, the probability of malingering according to OCT result was labeled as 1+, if it was between 50% and 25%, the probability was labeled as 2+ and so on. As a result, there were ten 1+, six 2+, eleven 3+ and six 4+ subjects with ODTP [Table 1].

Mean LogMAR VA increased significantly after SETs (LogMAR VAi: 1.07±0.59, LogMAR VA_aset_: 0.75±0.67, *P*<.001). There were 20 non-malingerers whose VA increased less than 2 Snellen lines or remained stable after SETs. The VAs of the remaining 13 of 33 (39%) subjects increased ≥2 Snellen lines after SETs [Table 1]. On the other hand, RNFL_temporal_ thickness reduction was predictive of non-malingering in most of the subjects. None of the 4 + subject (subjects with thinnest RNFL_temporal_ thickness category) was malingerer; however 80% of the 1+ subject (subjects with thicker RNFL_temporal_ thickness) were proved to be malingerer by means of SETs [Table 3]. All these malingerers confessed malingering after SETs

**Table 3:** The distribution of malingerers according to RNFL_temporal_ thickness.

RNFL_temporal_	Clinical diagnosis (SETs)	Total
result	Nonmalingerer	Malingerer
1+	2	8	10
2+	3	3	6
3+	9	2	11
4+	6	0	6
Total	20	13	33

SETs: Simulation examination techniques

**Figure 2:**
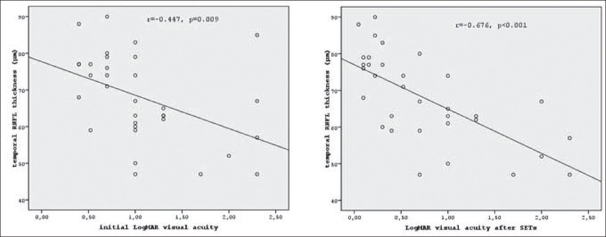
The correlations between temporal retinal nerve fiber layer thickness and visual acuity

The correlations between LogMAR-VAs (for both VA_i_ and VA_aset_) and RNFL_temporal_ thickness were significant. These significance and correlations for LogMAR-VA_aset_ were higher than the significance and correlation for LogMAR-VA_i_ [Fig. 2].

After these results, we decided to divide the subjects into two groups according to the RNFL_temporal_ thickness values. The subjects with a 1+ and 2+ RNFL_temporal_ thickness were labeled as RNFL_malingerer,_ the subjects with a 3+ and 4+ RNFL_temporal_ were labeled as RNFL_nonmalingerer._ because, we suggested that if a subject with thicker RNFL_temporal_ (1+ and 2+ subjects) should have higher VA than subjects with thinner RNFL_temporal_ (3+ and 4+ subjects). However, definite diagnosis of malingering was made by VA_aset_ after SETs as mentioned previously. Eleven of the 16 subjects with a 1+ and 2+ RNFL_temporal_ were malingerers, however only 2 of the 17 subjects with a 3+ and 4+ RNFL_temporal_ thickness were malingerers [Table 3]. The 1+ and 2+ subjects had significantly high ratio of malingering with respect to 3+ and 4+ subjects (*P*=0.001, chi-squared test).

Sensitivity of OCT-assessed RNFL_temporal_ thickness in detecting malingering was 11/13 (84.6%). Specificity, positive predictive value (PPV) and negative predictive value (NPV) were 15/20 (75.0%), 11/16 (68.8%), 15/17 (88.2%), respectively [Table 4].

**Table 4:** Calculation scheme for predictive value of PVEP

OCT result	Clinical evalution	Total
	Malingerer	Nonmalingerer	
RNFL_malingerer_	11 (True positive=a)	5 (false positive=b)	16
RNFL_nonmalingerer_	2 (False negative=c)	15 (True negative=d)	17
Total	13	20	33

Sensitivity:proportion of true positives (positive test result) among malingerers (a/a+c); Specificity: proportion of true negatives (negative test result) among nonmalingerers:d/b+d; Positive predictive value: proportion of true positives among all positive tests (a/a+b); Negative predictive value: proportion of true negatives among all negative tests:d/c+d

**Figure 3:**
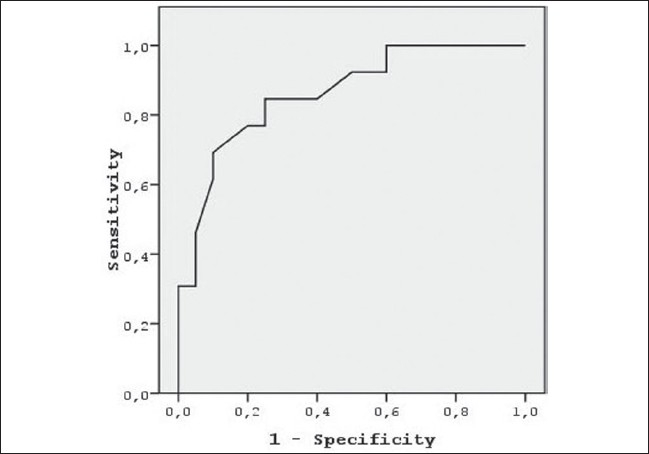
ROC curve of temporal retinal nerve fiber layer thickness for detection of malingering in subjects with optic disc temporal pallor

We used ROC curve analysis to find out the cut-off value of RNFL_temporal_ thickness for the prediction of malingering with the highest sensitivity and specificity. The cut-off value of RNFL_temporal_ thickness was 67.5 μm (sensitivity: 84.6%, specificity: 75.0%; AUC: area under the curve: 0.862, *P*=0.001, not shown in the Figure) [Fig. 3].

## Discussion

ODTP is an ophthalmoscopic finding mostly diagnosed in visual pathway pathology with various causes and in some other conditions such as myopic fundus.[20–24] Sometimes ODTP may be diagnosed in an otherwise normal ophthalmologic examination. One ophthalmologist may diagnose ODTP in a patient; however another may not in the same patient. Malingering and exaggerating any existing ophthalmologic pathology and its relation to medicolegal issues warrants having some objective criteria in diagnosing the disease truly. It's evident that diagnosis of malingering can be convincingly made in a case of no organic pathology including ODTP. However, this diagnosis is not straightforward in subjects with questionable pathology, and is not rare in demanding jobs like military, railways, roadways etc. In our study group, visual loss presented in the right eye in most (27 of 33 subjects; 81%), even in those with bilateral ODTP. This was most probably due to the traditional belief that right-hand-sided conscript who uses right eye for shooting would be unsuitable for the military. We were not able to find organic etiology for ODTP in our study group. We have to admit that we could not perform molecular analysis of hereditary or acquired disease in the differential diagnosis of ODTP, such as Leber's optic neuropathy.

Although OCT is accepted as an invaluable tool in the examination of the retina, variability in RNFL measurements, especially that in the quadrants, with Stratus OCT has been reported.[25] Age and ethnicity have been reported to influence RNFL thickness.[26–28] In a mean age of 44.5±16.1 years-old patient population from India, Sony *et al*.[28] found that age had a significant negative correlation with average RNFL thickness and with average superior and average inferior RNFL thickness; however, there was no significant correlation between age and average nasal or temporal RNFL thickness. In OCT measurements, axial length and refractive errors have to be considered.[29,30] We didn't examine axial length; however, both groups had similar distributions in refractive errors. Although our population seems to have higher RNFL_temporal_ thickness values, differences in normative database should be considered when evaluating the OCT results from different ethnicities. All the subjects and the controls were male young adults (ranging 19 to 27 years old) and from the similar environment in this study. In order to eliminate the bias in diagnosing we used strict criteria (described in the method section), and to decrease variability in RNFL measurements, we evaluated OCT recordings with signal strength of at least 8. All OCT procedures were performed by the experienced OCT technician during the same hours of the day, to exclude intraday variations among the subjects.

In this study, ROC curve showed that OCT has excellent diagnostic accuracy in defining ODTP; in other words, RNFL measurements in Stratus OCT may discriminate the malingerer from normal in subjects with ODTP. Using a single RNFL_temporal_ thickness cut-off value in the assessment of malingering in subjects with ODTP is, of course, not so logical. We think that if even present, using that single value without support of SETs may result in misdiagnosis of malingering. However, the ophthalmologists who come face to face with malingerers in their practice may benefit greatly from this objective value from OCT in their record for medicolegal issues. The relationship between RNFL_temporal_ thickness and VA has been shown.[16–19] In this study, we used SETs, which are accepted golden standard for diagnosing malingering. Since we aimed to have a cut-off value for diagnosing malingering in suspected malingerers with ODTP objectively, we compared RNFL_temporal_ thicknesses in eyes of malingerers diagnosed with SETs to those of age-and sex-matched healthy controls. The RNFL values of the other eyes with SA of 1.0 in either the involved eye (in bilateral cases) or uninvolved one in the study group were not considered for comparison. Although practically illogical to pretend as normal in our sample population, SA of 1.0 in one of the eye and decreased vision in the other in subjects with bilateral ODTP may suggest negative simulation. This, which was not in the scope of our study, also may complicate intraindividual comparison in the study group. In this study, we defined that RNFL_temporal_ thickness of 67.5 micrometer diagnose malingering with the highest sensitivity and specificity. This means that subjects with RNFL_temporal_ thickness below that value have significantly lower VAs than subjects with higher values. We found that the rate of the malingering decreased with thinning of RNFL_temporal_ thickness [Table 3]. The subjects with thicker RNFL_temporal_ were prone to malingering in the study population. It is plausible that the subjects with a thicker RNFL_temporal_ thickness (but still thinner than normal) have a less severe pathology, needing exaggeration in reaching secondary gain that is exclusion from the military service. However, subjects with thinner RNFL_temporal_ thickness have more severe disease or enough pathology for exclusion. In this study, we showed that while 69% of subjects with 1+ and 2+ RNFL_temporal_ were malingerers, this ratio was 12% in subjects with 3+ and 4+ RNFL_temporal_ thicknesses.

The sensitivity (84.6%) of OCT in detecting malingering was found to be higher than specificity (75.0%). This finding emphasizes that OCT-assessed RNFL_temporal_ assessment is valuable in detecting malingerers among real malingerers (or clinically proved malingerers by SETs) than in detecting non-malingerers among real nonmalingerers. It should not be forgotten that 25.0% of real nonmalingerers may be misdiagnosed as malingerers with this technique. In addition, NPV (88.2%) was higher than PPV (68.8%). This means that if OCT result predicts a subject as nonmalingerer, the probability of this subject to be a real nonmalingerer is 88.2%. It is apparent that if OCT result predicts a subject as malingerer, the probability of this subject being a real nonmalingerer is rather high, 31.2% (PPV: 68.8%). So, a negative OCT result (or a higher RNFL thickness) is a more valuable result than a positive (or a thinner RNFL thickness) result in discriminating malingering/nonmalingering. In this study, a true negative result does not mean the absence of optic nerve pathology. However, it means that the RNFL_temporal_ thickness in that subject is thin enough to explain the low VA. This study did not aim to find the significance of RNFL thickness in diagnosing optic nerve pathology. The result of this study has epidemiologic importance in a potentially malingering population, and has to be considered as supportive evidence for malingering in subjects with ODTP.

In a recent study, we have showed a considerable value of pattern visual evoked potentials (PVEP) to five check sizes in detecting malingering.[31] Apart from this study, PPV was more valuable than NPV in that study. It is apparent that using these two modalities together in detecting malingering in patients with ODTP may give more reliable results.

In conclusion, OCT-assessed RNFL thickness may be used as an adjunctive method in determining the existence of malingering in patients with ODTP.
